# Concerns about “Stress-Induced MazF-Mediated Proteins in Escherichia coli”

**DOI:** 10.1128/mBio.00825-19

**Published:** 2019-06-04

**Authors:** Joseph T. Wade, Michael T. Laub

**Affiliations:** a Wadsworth Center, New York State Department of Health, Albany, New York, USA; b Department of Biomedical Sciences, School of Public Health, University at Albany, Albany, New York, USA; c Department of Biology, Massachusetts Institute of Technology, Cambridge, Massachusetts, USA; d Howard Hughes Medical Institute, Massachusetts Institute of Technology, Cambridge, Massachusetts, USA; Northeastern University; Harvard University

**Keywords:** MazF, translation, toxin/antitoxin systems

## LETTER

In their recent study, “Stress-Induced MazF-Mediated Proteins in Escherichia coli,” Nigam et al. (1) identified 42 Escherichia coli proteins whose expression increased following induction of the MazF RNase toxin by nalidixic acid (NA). Of the 42 corresponding genes, 36 have an ACA sequence, the target site for MazF cleavage, <100 nt upstream of their start codon. Based on this observation, Nigam et al. tested whether NA-dependent changes in the expression of a green fluorescent protein (GFP) reporter were affected by the positions of upstream ACA sequences. The major conclusion drawn by Nigam et al. was that the presence of an ACA sequence <100 nt upstream of a start codon is associated with MazF-dependent regulation of expression for the corresponding gene. As described in detail below, none of the data presented by Nigam et al. support this conclusion.

As stated by Nigam et al. (1), ACA sequences are frequently found <100 nt upstream of genes in E. coli, as would be expected for any trinucleotide sequence. ACA sequences were found upstream of 36 of the 42 genes listed in Table 1 of the article by Nigam et al. While Nigam et al. describe this frequency as “remarkable,” it is anything but. In fact, for the 42 genes listed in their Table 1, the frequency of genes with an ACA trinucleotide <100 nt upstream is not significantly higher than that for the set of all E. coli genes (Fisher’s exact test, one tailed, *P = *0.18) or the control set of 2,807 genes described by Nigam et al. as having a “free region upstream” (Fisher’s exact test, one tailed, *P = *0.21). Thus, these data do not support the conclusion that upstream ACA sequences contribute to MazF-dependent regulation. Moreover, given that most 5′ untranslated region (UTR) lengths for E. coli genes are <50 nt (2), many of the ACA sequences listed in Table 1 of Nigam et al.’s article are expected to be located upstream of the transcription start site for the corresponding gene. For example, the *grpE*, *tyrB*, and *upp* 5′ UTRs are 39, 32, and 37 nt long, respectively (3–5), but the ACA sequences listed in Table 1 are 90, 74, and 81 nt upstream of the respective start codons and, hence, would not be present in the mRNAs.

Nigam et al. (1) used a *gfp* reporter system to show that the position of an ACA sequence upstream of the start codon affects the degree to which GFP expression is affected by NA treatment. While the differences observed in GFP expression are modest, the effect of NA does appear to be dependent upon ACA position relative to the start codon. However, this experiment lacks a critical control to show that the effects of NA treatment are dependent upon MazF. Specifically, Nigam et al. did not determine whether the effects of ACA location on NA-dependent changes in GFP expression were lost in a strain lacking *mazF*. Without this key control experiment, it is impossible to conclude anything about the role of MazF from these data, since NA treatment likely impacts transcription due to its effects on supercoiling (6). Moreover, even if one assumes that upstream ACA sequences do impact MazF-mediated regulation, the likelihood of the *gfp* reporter construct showing this effect is very low, since only 36 genes from a pool of thousands with upstream ACA sequences were found to be upregulated by MazF.

In summary, the data presented by Nigam et al. (1) do not support the conclusion that MazF cleavage at ACA sequences in mRNA 5′ UTRs leads to their increased translation. We propose that the genes described by Nigam et al. as being expressed more highly upon MazF induction are regulated indirectly as a consequence of widespread RNA processing by MazF. Consistent with this idea, for the 42 proteins identified by Nigam et al. as being upregulated by MazF, the corresponding genes are significantly enriched for genes whose expression is responsive to the stress-associated σ factors σ^38^ and σ^32^ (20/42 genes are upregulated upon σ^38^ expression [7], and 13/42 are transcribed by RNA polymerase associated with σ^32^ [8]; binomial-test *P = *1.6e−09). Lastly, we note that the notion of a “stress-induced translation machinery” has not withstood careful additional and independent scrutiny by the field (9, 10) and that these very relevant studies were not cited by Nigam et al.
